# Study Bioprospecting of Medicinal Plant Extracts of the Semiarid Northeast: Contribution to the Control of Oral Microorganisms

**DOI:** 10.1155/2012/681207

**Published:** 2012-06-06

**Authors:** Maria Suênia P. Silva, Deysiane O. Brandão, Thiago P. Chaves, Amaro L. N. Formiga Filho, Edja Maria M. de B. Costa, Vanda L. Santos, Ana Cláudia D. Medeiros

**Affiliations:** ^1^Laboratório de Desenvolvimento e Ensaio de Medicamentos, Universidade Estadual da Paraíba, 58.429-500 Campina Grande, PB, Brazil; ^2^Programa de Pós-graduação em Odontologia, Universidade Estadual da Paraíba, 58.429-500 Campina Grande, PB, Brazil

## Abstract

Dental pathologies can be caused by plaque-forming bacteria and yeast, which reside in the oral cavity. The bacteria growing in dental plaque, a naturally occurring biofilm, display increased resistance to antimicrobial agents. The objective was the evaluation of a preclinical assay of medicinal plants of the semiarid region from the northeast against oral pathogenic microorganism, aiming at bioprospecting a new product. The selection of plant material for this study was based on the ethnobotanical data on the traditional use of plants from the semiarid region. The thirty extracts were subjected to the determination of antibiofilm activity against gram-positive, gram-negative bacteria and yeast. The hydroalcoholic extract which showed positive antibiofilm activity against most of the microorganisms tested in agar diffusion assay was further tested for the determination of minimum inhibitory concentration (MIC) and Bioassay with *Artemia salina*. Plant samples tested in this study exhibited good antibiofilm activity for the treatment of oral problems. The *Schinopsis brasiliensis* showed greater activity for *Pseudomonas aeruginosa* and *Staphylococcus aureus*, but toxicity against *Artemia salina*.

## 1. Introduction

Dental caries, periodontal disease, and candidiasis are important dental pathologies affecting humankind. These conditions are caused by plaque-forming bacteria and yeast, which reside in the oral cavity. Periodontal diseases have mainly been associated with *Actinomyces*, *Actinobacillus*, *Streptococcus*, and *Candida *species [1]. It is generally recognized that the bacterial growth in biofilms imparts a substantial decrease in susceptibility to antimicrobial agents compared with cultures grown in suspension. It is therefore not surprising that bacteria growing in dental plaque, a naturally occurring biofilm, display increased resistance to antimicrobial agents [2].

The resistance development by pathogens to the existing pharmaceutical has led to the intensification of the search for novel drugs, against fungal, parasitic, bacterial, and viral infections. Plant-derived antimicrobials have a long history of providing the much needed novel therapeutics. A number of traditionally used medicinal plants have to date been screened for various biological activities in both *in vivo* and *in vitro* models. The chemical investigation from medicinal plants purported to have medicinal properties has yielded numerous purified compounds which have proven to be indispensable in the practice of modern medicine [3].

Brazil has a great potential for biodiversity and wealth of traditional knowledge accumulated by local people who have direct access to nature and the products of biodiversity. In this sense, the Caatinga, main semiarid's biome, represents the fourth largest area covered by a single vegetation form in Brazil, accounting for about 60% of the northeast territory. There has been increasing interest in acquiring knowledge regarding the medicinal plants within the Caatinga area, and some publications describe the rich flora of this region as having many medicinal purposes [4].

This work aims at the evaluation of a preclinical assay of medicinal plants from the folk medicine of the semiarid region of the northeast against oral pathogenic microorganisms, in order to bioprospect a new product.

## 2. Materials and Methods

### 2.1. Plant Materials

The medicinal plants were collected from the semiarid region in the State of Paraíba. Voucher specimen were prepared and identified at the Professor Jayme Coelho de Morais Herbarium (PRU), Federal University of Paraiba and at the Dárdamo de Andrade Lima herbarium Agronomic Institute of Pernambuco (Table 1).

### 2.2. Extracts Preparation

The plant material was air dried at 40°C and ground to a fine powder. The powdered material (200 g) was extracted with 1000 mL ethanol/distilled water with 10, 20, 30, 50, and 70% (v/v) for 5 days with occasional stirring, accordingly named as HEx1, HEx2, HEx3, HEx4, and HEx5, respectively, and stored at room temperature in amber flasks.

### 2.3. Antibiofilm Activity

 The microorganisms used in this study included *Pseudomonas aeruginosa* (ATCC 27853), *Streptococcus mutans* (ATCC 25175), *Streptococcus salivarius* (ATCC 7073), *Streptococcus oralis* (ATCC 10557), *Lactobacillus casei* (ATCC 7469), *Enterococcus faecalis* (ATCC 29212), *Staphylococcus aureus* (ATCC 25923), *Candida albicans* (ATCC 18804), *Candida tropicalis* (ATCC 13803), *Candida krusei* (ATCC 34135). Bacteria were grown in the Mueller Hinton agar medium with 5% blood agar under anaerobic conditions in anaerobic jar, at 37°C for 48 h. Sabouraud Dextrose Agar medium was used for culturing *Candidas* at 25°C for 24 h under aerobic conditions.

#### 2.3.1. Cylinder-Plate Assay

The microorganisms were individually inoculated into tubes containing 5 mL sterile 0.9% saline solution. The suspension was adjusted spectrophotometrically at 625 nm which is equivalent to 10^6^ CFU/mL. The agar was composed of 2 separate layers, and only the upper layer was inoculated. The Mueller Hinton agar (20 mL) was poured into microbiological Petri dish (100 mm × 20 mm) for the base layer. After solidification, the other layer containing 5 mL inoculated medium, with the different microorganisms, was distributed to the surface of each base layer. For the 5 × 1 design, six stainless-steel cylinders of uniform size (8 mm × 6 mm × 10 mm) were placed on the surface of inoculated agar, and an exactly measured volume (100 *μ*L) of the corresponding extract was deposited into five cylinders, while the other one was filled with either the standard solution. In the bioassay, ten plates were prepared. All plates were left for 2 hour at room temperature and then incubated at 37°C ± 1°C (for bacteria) for 24 h and 30°C ± 1°C (for yeast) for 24 h. The diameter (in mm) of growth inhibition zones was carefully measured using a caliper with at least 0.1 mm accuracy.

#### 2.3.2. Minimal Inhibitory Concentration (MIC) Determination

The assay was performed by the broth microdilution method. It was performed in 96 well microplates according to the Clinical and Laboratory Standards Institute [27] procedures. The inoculums were standardized in tubes containing 5 mL sterile 0.9% saline solution. The suspension was adjusted spectrophotometrically at 625 nm which is equivalent to 10^6^ CFU/mL. One hundred microlitres of each hydroalcoholic extract were serially diluted threefold with sterile Mueller Hinton broth or with sterile Sabouraud Dextrose broth in a 96-well microlitre plate for each microorganisms strains studied. A similar threefold serial dilution of chlorhexidine was used as a positive control against each bacterium and nystatin against each *Candida*. One hundred microlitres of each microorganism culture were added to each well. Ethanol/water was included as negative controls. The plate incubated at 37°C ± 1°C (for bacteria) for 24 h and 30°C ± 1°C (for yeast) for 24 h. Bacterial growth was indicated by adding 20 *μ*L of aqueous solution of resazurin (Sigma-Aldrich) to 0.01% with further incubation at 37°C ± 1°C for 2 h. Viable bacteria reduced the yellow dye to a pink color. The MIC was defined as the lowest concentration that inhibited the colour change of resazurin.

### 2.4. Bioassay with *Artemia salina*

This bioassay was performed with extracts that showed antibiofilm activity to evaluate the EtOH extract toxicity; it used the brine shrimp (*Artemia salina*) lethality test. The 50 g of eggs of *A. salina *were incubated in sea water (pH 8 ± 0,5 and 28°C) at artificial light during 24–36 hours for cysts occlusion and larvae obtaining. After hatching, they were collected and put into tubes containing different EtOH extract concentrations (2000, 1500, 1000, 500, and 250 *μ*g/mL), and the blank control was always conduced. The set was incubated at artificial light for 24 h, and then the survivor larvae were counted to determine the LC50 using the Probit method. The bioassay was repeated three times. As the measure of extract toxicity, the LC50 value lower than 1000 *μ*g/mL is considered bioactive [28].

### 2.5. Statistical Analysis

The results were expressed as mean. Differences among means were statistically compared using the Student's *t*-test for antibiofilm assay. The values were considered significantly different when *P* < 0.05. The software used was the Origin software. The LC50 for Bioassay with *A. salina* was determined according to the Probit statistical method using the software STATPLUS 2005.

## 3. Results and Discussion

### 3.1. Antibiofilm Activity

The selection of plant material for this study was based on the ethnobotanical data on the traditional use of plants from the semiarid region. The thirty extracts were subjected to the determination of antibiofilm activity against gram-positive, gram-negative bacteria and yeast (Table 2). The extracts of *H. mutabilis* and* A. coriacea *presented no inhibitory activity against the microorganisms tested, but Toledo et al. [29] showed that the extract obtained from *A. coriacea* showed the best activity against *Candida albicans* and *C. parapsilosis*. All extracts exhibited antibacterial activity against *S. oralis*. The growth of *S. mutans, S. salivarius, L. casei, C. albican, C. tropicalis*, and *C. krusei* was not inhibited by any concentration of extracts tested.

The *A. hispidum *extract presented activity only against *S. oralis. *The study conducted by Ederwor and Olajire [30] confirms the antimicrobial activity of the extracts of *P. aeruginosa and S. aureus*.

The *X. americana* bark extract showed activity against *E. faecalis, S. aureus*, and *S. oralis.* The zones of inhibition observed at *E. faecalis* were statistically different compared with those of chlorhexidine (positive control). And for another bacterium, the chlorhexidine presented bigger inhibition halo than that promoted by the *E. faecalis *and *S. aureus*. Costa et al. [31] demonstrated the antimicrobial activity of the *X. americana* bark extracts at *E. faecalis* and showed that they may represent alternative substances in endodontic treatment. And in another study Falcão and Menezes [32] observed that *C. albicans *was the least susceptible among the tested yeast.

The best results were obtained with *S. brasiliensis *extract against *S. aureus.* This extract presents higher efficacy in the HEx5 for *P. aeruginosa, *Hex1–Hex4 for *S. aureus*, and Hex1–Hex3 for *S. oralis* compared with chlorhexidine. This result was consistent with that discussed by Goleniowski et al. [14] and de Barros et al. [15]. This confirmed that antibiofilm activity can be related to the higher proportion of tannins and flavonoids in the plant like the observed by Siqueira et al. [33].

The hydroalcoholic extract which showed positive antibiofilm activity against most of the microorganisms tested in agar diffusion assay was further tested for the determination of minimum inhibitory concentration (MIC) and Bioassay with *Artemia salina*.

### 3.2. Minimal Inhibitory Concentration (MIC) Determination

Minimum inhibitory concentrations (MICs) of extracts for antibiofilm activity were determined using the microdilution bioassay as described by CLSI [27] (Table 3).

Studies showed that the MIC values indicate that *S. brasiliensis* extract was more efficient, corroborating the results of the previous experiment. The important activity on *S. aureus *confirms the best activity obtained in cylinder-plate assay, which revealed this strain as one of the most susceptible with MIC ranging from 0.250 to 0.063 *μ*L/*μ*L. The *S. oralis* strain was the least sensitive (MIC = 1.000 *μ*L/*μ*L). The *P. aeruginosa* HEx1 was the most sensitive (MIC = 0.004 *μ*L/*μ*L). Similar result was found by Goleniowski et al. [14] and de Barros et al. [15].

 The *X. americana *extract presented activity against *S. aureus *(MIC = 0.250–0.063 *μ*L/*μ*L), *E. faecalis* (MIC = 1.000 *μ*L/*μ*L), and *S. oralis* (MIC = 1.000 *μ*L/*μ*L). Omer and Elnima [34] reported activity of the methanolic and aqueous extract against *S. aureus.* Koné et al. [35] demonstrated that the ethanolic root extract exhibited some activity against *S. aureus *and *E. faecalis.*

### 3.3. Bioassay with *Artemia salina*

Determination of the toxicity to *Artemia salina *has been used efficiently to analyze the biological potential of plant extracts. Several natural compounds, particularly substances with antitumoral, antimicrobial, insecticidal, and antitrypanosomal activity, have been tested through these bioassays showing significant correlation [36].

The results showed that the extract of *A. hispidum* presented LC50 = 4.101 *μ*g/mL, *S. brasiliensis *LC50 = 428 *μ*g/mL, and *X. americana *LC50 = 4.262. Meyer et al. [28] described a lethal concentration (LC50) based on the toxicity of substances to the larvae of *A. salina*. According to the scale, LC50 values < 500 *μ*g·mL^−1^ indicate toxicity, LC50 between 500 and 1000 *μ*g·mL^−1^ denote moderate toxicity, while LC50 > 1000 *μ*g·mL^−1^ suggest lack of toxicity.

In this work *S. brasiliensis *showed data that indicate toxicity. The study conducted by de Barros et al. [15] confirms the possible plant toxicity. It was related that this can be explained by the high concentration of polyphenols (tannin, flavonoids, and other phenolics compounds) present in this plant and which are well known for their toxicity against *A. salina.*

## 4. Conclusion

 Plant samples tested in this study exhibited good antibiofilm activity for the treatment of oral problems. The *S. brasiliensis *showed higher activity for *P. aeruginosa* and *S. aureus, *but toxicity against *Artemia salina. *The result suggests that studies to isolate and characterize the antibiofilm compounds of *S. brasiliensis *Engl, whereas their high antibacterial activity could be the base of development of new phytotherapic product with broad spectra. Studies *in vivo* of toxicity are essential.

This reinforces the concept that the ethnobotanical investigation of used plants is necessary since it will reveal a great number of positive responses in *in vitro* tests.

## Figures and Tables

**Table 1:** Dada ethnobotanical investigated plants.

Scientific name	Popular name	Family	Part used	Herbarium number	Popular use	Reference
*A. hispidum* DC.	Carrapicho-de-cigano	Asteraceae	EP	EAN-10957	Cough, respiratory problem, diuretic, diarrhea, sinusitis, malaria, infection in general, bronchitis and intestinal problems, typhus.	[4–11]

*A. coriacea* L.	Araticum	Annonaceae	SB	IPA21443	Stomach problems, diarrhea, and malaria.	[12, 13]

*H. mutabilis* Briq.	Alfavaca-de-caboclo	Lamiaceae	L	EAN-10699	Stomach problems, sudorific, expectorant, calmative, uterine inflammation, gastric ulcer, influenza, fever, cough, headache, rheumatism.	[6, 10, 14–18]

*S. brasiliensis* Engl.	Braúna	Anacardiacea	SB	EAN-14049	Cough, influenza, diarrhea, fractures, sexual impotence.	[5, 6, 10, 19, 20]

*X. americana* L.	Ameixa	Olacaceae	SB	EAN-100493	Antiseptic, malaria, toothache, skin urinary infection, diarrhea, asthma, anemia, inflammation, fibroids, sleep diseases, ulcer, gastritis, Fractures, cancer, itching.	[6, 21–26]

R: root; SB: stem-bark; L: leaves; EP: entire plant.

**Table 2:** Antimicrobial activity of plants tested against oral microorganisms.

Hydroalcoholic extract	Zones of inhibition (mm)									
	Bacterial strains							Fungal strains		
	P.a.	E.f.	S.a.	S.m.	S.o.	S.s.	L.c.	C.a.	C.t.	C.k.
*Acanthospermum hispidum *										
HEx1	0.0	0.0	0.0	0.0	11.7	0.0	0.0	0.0	0.0	0.0
HEx2	0.0	0.0	0.0	0.0	11.6	0.0	0.0	0.0	0.0	0.0
HEx3	0.0	0.0	0.0	0.0	11.9	0.0	0.0	0.0	0.0	0.0
HEx4	0.0	0.0	0.0	0.0	11.7	0.0	0.0	0.0	0.0	0.0
HEx5	0.0	0.0	0.0	0.0	0.0	0.0	0.0	0.0	0.0	0.0
*Ximenia americana*										
HEx1	0.0	0.0	13.3	0.0	0.0	0.0	0.0	0.0	0.0	0.0
HEx2	0.0	0.0	13.3	0.0	0.0	0.0	0.0	0.0	0.0	0.0
HEx3	0.0	14.1	14.9	0.0	0.0	0.0	0.0	0.0	0.0	0.0
HEx4	0.0	0.0	13.3	0.0	0.0	0.0	0.0	0.0	0.0	0.0
HEx5	0.0	0.0	12.6	0.0	11.2	0.0	0.0	0.0	0.0	0.0
*Schinopsis brasiliensis*										
HEx1	17.3	0.0	25.1	0.0	14.7	0.0	0.0	0.0	0.0	0.0
HEx2	16.6	0.0	27.9	0.0	14.8	0.0	0.0	0.0	0.0	0.0
HEx3	16.9	10.8	33.2	0.0	14.0	0.0	0.0	0.0	0.0	0.0
HEx4	17.0	10.3	30.4	0.0	10.5	0.0	0.0	0.0	0.0	0.0
HEx5	18.0	0.0	12.4	0.0	11.2	0.0	0.0	0.0	0.0	0.0
Chlorhexidine	19.7	13.4	16.0	13.0	12.6	12.5	12.3	—	—	—
Nistatine	—	—	—	—	—	—	—	23.2	22.0	23.2

P.a.: *P. aeruginosa; *E.f.: *E. faecalis; *S.a.: *S. aureus*; S.m.: *S. mutans*; S.o.: *S. oralis*; S.s.: *S. salivarius;* L.c.: *L. Casei*; C.a.: *C. albicans*; C.t.: *C. tropicalis*; C.k.: *C. krusei*.

**Table 3:** Mean MIC results of selected plants on oral microorganisms.

Hydroalcoholic extract	MIC (*μ*L/*μ*L)			
	Microorganisms tested			
	P.a.	E.f.	S.a.	S.o.
*Acanthospermum hispidum *				
HEx1	—	—	—	1.000
HEx2	—	—	—	1.000
HEx3	—	—	—	1.000
HEx4	—	—	—	1.000
*Ximenia americana*				
HEx1	—	—	0.063	—
HEx2	—	—	0.063	—
HEx3	—	1.000	0.063	—
HEx4	—	—	0.250	—
HEx5	—	—	0.250	1.000
*Schinopsis brasiliensis*				
HEx1	0.004	—	0.063	0.500
HEx2	0.500	—	0.063	1.000
HEx3	1.000	1.000	0.063	0.500
HEx4	0.063	1.000	0.063	0.500
HEx5	0.063	—	0.063	0.500

P.a.: *P. aeruginosa; *E.f.: *E. faecalis; *S.a.: *S. aureus*; S.o.: *S. oralis*.
